# Causal modeling reveals cell–cell communication dynamics in the tumor microenvironment during anti-PD-1 therapy in breast cancer patients

**DOI:** 10.1093/bib/bbag139

**Published:** 2026-04-19

**Authors:** Aodong Qiu, Han Zhang, Joseph D Ramsey, Bryan Andrews, Boyang Sun, Shuangxia Ren, Mengyao Lu, Kun Zhang, Gregory F Cooper, Binfeng Lu, Lujia Chen, Xinghua Lu

**Affiliations:** Department of Biomedical Informatics, University of Pittsburgh, 5607 Baum Blvd, Pittsburgh, PA, 15206, United States; School of Medicine, Tsinghua Medicine, Tsinghua University, No. 30, Shuangqing Road, Haidian District, Beijing, 100084, China; Department of Biomedical Informatics, University of Pittsburgh, 5607 Baum Blvd, Pittsburgh, PA, 15206, United States; Department of Philosophy, Carnegie Mellon University, 5000 Forbes Avenue, Pittsburgh, PA, 15213, United States; Department of Psychiatry & Behavioral Sciences, University of Minnesota, 2312 S 6th St, Minneapolis, MN, 55454, United States; Mohamed bin Zayed University of Artificial Intelligence, Building 1B, Masdar City, Abu Dhabi, United Arab Emirates; Department of Biomedical Informatics, University of Pittsburgh, 5607 Baum Blvd, Pittsburgh, PA, 15206, United States; Department of Biomedical Informatics, University of Pittsburgh, 5607 Baum Blvd, Pittsburgh, PA, 15206, United States; Department of Philosophy, Carnegie Mellon University, 5000 Forbes Avenue, Pittsburgh, PA, 15213, United States; Mohamed bin Zayed University of Artificial Intelligence, Building 1B, Masdar City, Abu Dhabi, United Arab Emirates; Department of Biomedical Informatics, University of Pittsburgh, 5607 Baum Blvd, Pittsburgh, PA, 15206, United States; Center for Discovery and Innovation, Hackensack Meridian Health, 111 Ideation Way, Nutley, NJ, 07110, United States; Department of Biomedical Informatics, University of Pittsburgh, 5607 Baum Blvd, Pittsburgh, PA, 15206, United States; Department of Biomedical Informatics, University of Pittsburgh, 5607 Baum Blvd, Pittsburgh, PA, 15206, United States; Department of Pharmaceutical Sciences, University of Pittsburgh, 3501 Terrace St, Pittsburgh, PA, 15213, United States

**Keywords:** cell–cell communication, causal inference, anti-PD-1 treatment, tumor microenvironment, breast cancer

## Abstract

Immune checkpoint blockade (ICB) targeting PD-1/PD-L1 axis has transformed breast cancer treatment, yet how therapy reshapes the tumor microenvironment (TME) through cell–cell communication (CCC) remains unclear. Existing CCC inference methods relying on correlations have difficulty distinguishing genuine signaling from confounded associations. Here, we present a causal inference framework that uses single-cell data and leverages treatment as an instrumental variable to identify genuine CCC networks, referred to as scIVCCC, which infers causal signal transduction across cell types. Applying scIVCCC to single-cell RNA-seq data from 31 breast cancer patients before and after anti-PD-1 therapy, we constructed causal CCC networks linking exhausted T cells to tumor-associated macrophages (TAMs). Our analysis reveals a dual role of T cell-macrophage crosstalk: CD4+ and CD8+ exhausted T cells drive anti-tumor M1-like TAMs activation via TNF–TNFRSF1A, TNFSF14–LTBR, and ICAM1–ITGAL/ITGB2. Conversely, they also induce immunosuppressive M2-like polarization through pathways such as TNF–TNFRSF1B (TNFR2), TNFSF14–TNFRSF14 (HVEM), and RPS19–C5AR1, which likely contribute to therapeutic resistance. Our causal modeling suggests that receptors within these networks, such as C5AR1, TNFR2, and CSF1R, may serve as potential candidates for combination therapies to enhance anti-PD-1 efficacy. Collectively, these findings demonstrate that scIVCCC offers a robust framework for dissecting treatment-induced CCC dynamics and prioritizing actionable targets for clinical translation.

## Introduction

Breast cancer remains the most common malignancy affecting women worldwide, characterized by diverse biological behaviors and responses to treatment across its subtypes [1]. Among breast cancer subtypes, triple-negative breast cancer (TNBC) is notably aggressive and lacks targeted therapies due to its absence of estrogen and progesterone receptors and HER2 expression [2]. Historically, breast cancer has not been considered highly immunogenic, but in recent decades, discoveries have shown substantial tumor-infiltrating lymphocytes (TILs) in some subtypes, especially in TNBC, suggesting potential responsiveness to immunotherapies [3, 4].

Immune checkpoint blockade (ICB) therapies have revolutionized cancer treatment by targeting regulatory pathways in T cells to enhance the immune system’s response against cancer cells [5]. Agents targeting the Programmed Death-1 (PD-1) pathway, which is often exploited by tumors to evade anti-tumor immune responses, have shown promising results in various cancers, including breast cancer [6]. Despite these advances, the immunotherapy response rate in breast cancer remains modest. In TNBC, the most immunogenic subtype, the response rate to anti-PD-1 monotherapy is generally below 20% [7]. The precise mechanisms by which ICB therapies modify the tumor microenvironment (TME) and promote anti-tumor activity remain incompletely understood. The complexity of the TME, which includes cancer cells, immune cells, stromal cells, the extracellular matrix, and soluble factors, makes it difficult to fully comprehend how these therapies alter cellular communication and the mechanisms of treatment resistance [8].

As reported by Cesaro et al. [9], the field of cell–cell communication (CCC) inference has expanded rapidly, with over 100 bioinformatics tools developed in recent years employing diverse computational strategies, including statistics-based, network-based, correlation-based, machine learning, and matrix decomposition approaches. Widely used tools such as CellChat [10], NicheNet [11], and CellPhoneDB [12] have been applied to characterize ligand-receptor-mediated signaling across diverse cell types. However, these tools fundamentally compute ‘intercellular scores’ representing statistical evidence of signaling between subpopulations of cells, based on expression patterns and prior knowledge networks [9]. Consequently, they often do not explicitly distinguish causal regulatory effects from confounded associations, nor are they suitable for inferring intercellular influence driven by specific perturbations, such as ICB treatment. This highlights the need for causal inference frameworks to uncover communication channels that likely mediate therapeutic responses.

**Figure 1:**
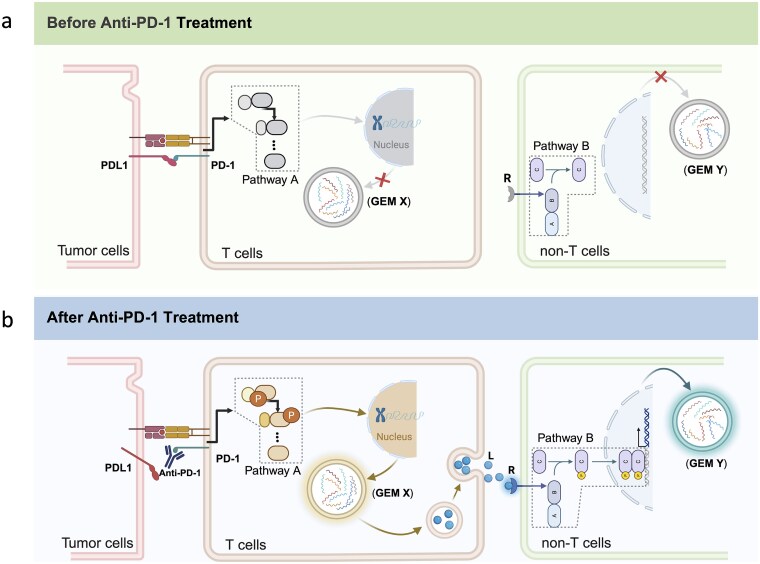
**Conceptual overview of the cell–cell communication mechanism and the causal inference framework. a**, Before anti-PD-1 treatment. The PD-1/PD-L1 interaction between T cells and tumor cells suppresses T cell signaling (Pathway A), resulting in minimal expression of gene expression module *X* (GEM *X*) and limited ligand secretion. Non-T cells remain in a basal state with inactive pathway B and minimal GEM Y expression. **b**, After anti-PD-1 treatment. The therapeutic antibody blocks PD-1/PD-L1 interaction, releasing T cells from suppression. Activated pathway A drives GEM *X* expression and ligand (L) secretion. Ligands bind to receptors (R) on non-T cells, activating pathway B and inducing GEM *Y* expression. This illustrates the biological mechanism underlying treatment-induced cell–cell communication.

To illustrate the biological rationale for a causal approach, consider the mechanism of anti-PD-1 therapy (Fig. 1). Prior to treatment, PD-1/PD-L1 engagement between T cells and tumor cells suppresses intracellular T cell signaling (Pathway A), resulting in diminished expression of downstream gene expression modules (GEMs) comprising co-regulated differentially expressed genes (DEGs) and limited ligand secretion. Consequently, ligand-receptor (LR)-mediated communication with non-T cells remains minimal, and downstream pathways (Pathway B) along with their associated GEMs are not activated (Fig. 1a). However, following anti-PD-1 treatment, therapeutic blockade of the PD-1/PD-L1 interaction releases T cells from immunosuppression, activating Pathway A and inducing the expression of DEGs within GEM X (Fig. 1b). Activated T cells subsequently secrete ligands that bind cognate receptors on non-T cells, triggering Pathway B and driving DEG expression in GEM Y. This sequential cascade, wherein treatment-induced T cell activation propagates to non-T cell populations through LR-mediated intercellular communication, establishes a causal chain.

To address this challenge, we developed the single-cell Instrumental Variable analysis for Cell–Cell Communication (scIVCCC), a causal inference framework that leverages treatment as an instrumental variable and applies conditional independence testing to identify mediating LR pairs, enabling the distinction of genuine cell–cell communication from confounded associations. Using this framework, we analyzed single-cell RNA sequencing data from 31 breast cancer patients receiving anti-PD-1 treatment (pembrolizumab) reported by Bassez *et al* [4], comparing pre- and on-treatment conditions. We examined treatment-induced transcriptional changes across diverse cell populations and employed instrumental variable analysis combined with conditional independence testing to construct a comprehensive map of the CCC network influenced by anti-PD-1 therapy. Through this approach, we identified pivotal ligand-receptor pairs that mediate communication among various cell types, with particular focus on the interactions between exhausted T cells and tumor-associated macrophages (TAMs). Notably, while we demonstrate this framework in the context of anti-PD-1 therapy in breast cancer, the methodology is broadly applicable to any perturbation-based study where treatment effects propagate through intercellular communication, including other immunotherapies, targeted therapies, and disease contexts beyond oncology.

These findings advance our understanding of TME remodeling during immunotherapy and offer potential biomarkers for predicting treatment response. By distinguishing causal regulatory effects from spurious associations, this study prioritizes high-confidence molecular targets for experimental validation and potential translation into clinical applications, bridging the gap between computational discovery and therapeutic intervention.

## Methods

### Overview of the scIVCCC framework

**Figure 2:**
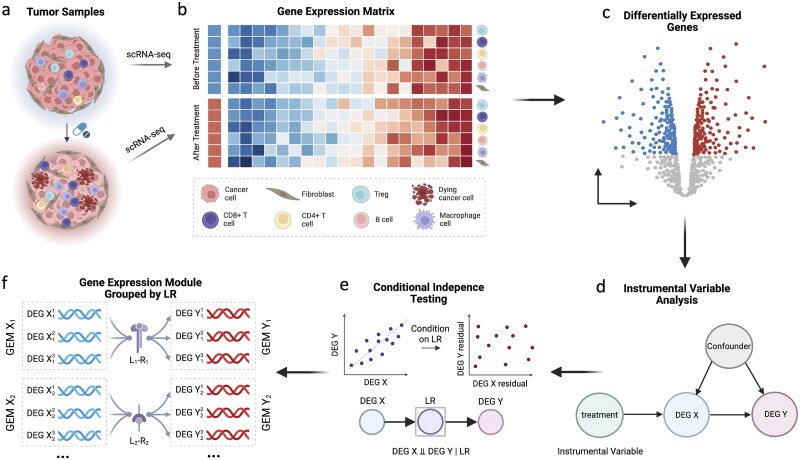
**Overview of the scIVCCC analytical framework.** Schematic illustration of the scIVCCC pipeline for inferring treatment-induced cell–cell communication networks. **a**, Paired tumor samples are collected from the same patients before and after treatment and undergo single-cell RNA sequencing. **b**, Gene expression matrices are generated for each condition across diverse cell types. **c**, Differentially expressed genes (DEGs) are identified by comparing the expression profiles of cells before and after treatment within each cell type. **d**, Instrumental variable analysis is then applied to identify causal relationships between DEGs from treatment-targeted cells (DEG *X*) and non-targeted cells (DEG *Y*), using treatment as an instrument to account for potential confounders. **e**, Subsequently, conditional independence testing (CIT) is performed to identify ligand-receptor (LR) pairs that mediate intercellular communication. If an LR pair mediates the DEG *X*–DEG *Y* relationship, conditioning on the LR interaction renders DEG *X* and DEG *Y* statistically independent (DEG *X*  $\bot\!\!\!\!\bot $ DEG *Y* | LR). **f**, DEG pairs sharing common LR mediators are grouped into gene expression modules, with sender cell DEGs forming GEM *X* and receiver cell DEGs forming GEM *Y*, yielding a mechanistically interpretable map of treatment-induced cell–cell communication networks. The notation DEG ${X}_k^i$ denotes the *i*-th DEG in the sender cell module associated with the *k*-th LR pair (L*_k_*–R*_k_*), and similarly DEG ${Y}_k^j$ denotes the *j*-th DEG in the corresponding receiver cell module.

We developed scIVCCC, a causal inference framework for identifying treatment-induced cell–cell communication networks from single-cell transcriptomic data (Fig. 2). The framework requires paired tumor samples collected from the same patients before and after a perturbation that directly targets a specific cell population (Fig. 2a). Single-cell RNA sequencing is performed on both conditions to capture transcriptomic profiles across diverse cell types within the tumor microenvironment.

Following standard quality control, normalization, and cell type annotation, cell-type-specific expression matrices are generated (Fig. 2b). DEGs are identified using paired statistical tests comparing the expression before and after treatment (Fig. 2c). Many DEGs from different cell types are highly correlated. To distinguish genuine treatment-induced cell–cell communication from confounded correlations, instrumental variable (IV) analysis is employed (Fig. 2d). Specifically, let DEG *X* denote a DEG from the treatment-targeted cell type and DEG *Y* denote a DEG from a non-targeted cell type. The treatment serves as an instrumental variable that directly affects DEG *X*, influences DEG *Y* only through DEG *X* given that non-targeted cells lack the therapeutic target, and is independent of unmeasured confounders. DEG pairs passing the IV test are retained as candidate causal relationships.

Subsequently, conditional independence testing (CIT) is applied to identify LR pairs mediating intercellular signal transduction (Fig. 2e). If an LR pair mediates the relationship between DEG *X* and DEG *Y*, conditioning on the LR interaction renders DEG *X* and DEG *Y* statistically independent (DEG *X*  $\bot\!\!\!\!\bot $ DEG *Y* | LR). LR pairs satisfying this criterion, together with co-expression constraints requiring ligand co-expression with DEG *X* in sender cells and receptor co-expression with *Y* in receiver cells, are identified as candidate communication mediators. Finally, DEG pairs sharing common LR mediators are grouped into gene expression modules, with co-regulated DEGs from sender cells forming GEM *X* and those from receiver cells forming GEM *Y* (Fig. 2f). This modular organization yields a mechanistically interpretable map of treatment-induced cell–cell communication.

### Data collection and primary process of single-cell data

We collected the single-cell RNA-seq and T-cell receptor sequencing (TCR-seq) data of 31 early-stage breast cancer patients from the study NCT03197389. We used Scanpy version 1.11.0 [13] to process single-cell data. After the aggregation and normalization, we removed the cells expressing fewer than 500 genes or more than 5000 genes and the cells containing fewer than 400 UMIs (unique molecular identifiers) or more than 25 000 UMIs. Then, we filtered the cells with a high percentage (>20%) of mitochondrial genes. After filtration, we retained 134 752 cells in total. We used unsupervised cell clustering methods for feature reduction. Cells were divided into 22 clusters and then annotated as five cell types according to the cell type markers expression levels (Supplementary Fig. 1). We applied PCA [14], neighbor detection [15], and the Uniform Manifold Approximation and Projection (UMAP) [16] algorithms to achieve dimensionality reduction.

### Identify differentially expressed genes

To identify DEGs, we analyzed paired gene expression data from 31 patients, each with samples collected before and after treatment. Single-cell RNA sequencing data were aggregated into pseudo-bulk profiles and normalized using log2(TPM + 1) transformation. To account for inter-patient variability, we performed paired t-tests for each gene, comparing expression levels between the two conditions within each patient. Genes with a *P* < .05 and an absolute log2 fold-change greater than 0.5 were considered significant. For visualization purposes, volcano plots highlight genes meeting a more stringent threshold of *P* < .01.

### Using instrumental variable analysis to remove the effect of confounders

We used the R package ivreg [17] (version 0.6–3) for instrumental variable (IV) analysis to control for confounders. IV is implemented by using two-stage least-squares (2SLS) estimation [18]. In the first stage, we regress the endogenous variable *X* on the instrumental variable *Z* (treatment) to obtain the predicted values $\hat{X}$.


$$ \hat{X}=Z\hat{\delta}=Z{\left({Z}^TZ\right)}^{-1}{Z}^TX={P}_ZX $$

Where $\hat{\delta}$ is the estimated coefficient from the regression of *X* on *Z.* P_Z_ is the projection matrix formed from *Z*. After this step, $\hat{X}$ is correlated with *Z* but not correlated with the error of this regression. In the second stage, we use the $\hat{X}$ to perform the regression to *Y*.


$$ Y={\beta}_0+{\beta}_1\hat{X}+u $$

Where ${\beta}_0$ and ${\beta}_1$ are estimated coefficients, and $u$ is the error.

To apply IV analysis, three conditions must be met: relevance, exogeneity, and exclusion restriction. The instrumental variable *Z* must be correlated with the endogenous explanatory variable (X), uncorrelated with the outcome variable *Y* except through *X*, and uncorrelated with any confounders between *X* and *Y*. In our study (Fig. 5b), *Z* represents the anti-PD-1 treatment, *X* refers to the DEGs in *PDCD1*+ T cells or their subtypes, and *Y* corresponds to other cell-type DEGs. Since the treatment directly targets *PDCD1*+ T cells, *Z* is correlated with *X*. We calculated the residuals of *Y* regressed on *X* and filtered *X-Y* pairs where *Z* was uncorrelated with the residuals of *Y*, indicating that *Z* only affects *Y* through *X*. After confirming that our data met the IV assumptions, we grouped all cells by sample_id to generate pseudo-bulk data. We created a matrix with the expression levels of DEGs from both cell types (DEG *X* and *Y*) as columns and sample_ids as rows, adding a treatment column (0, before treatment, 1: after treatment). Applying ivreg to this matrix, we collected *P*-values for the IV test and filtered out DEG pairs with *P* > .05.

### Fisher-z conditional independence test

We applied the Fisher-z conditional independence test implemented in the causal-learn [19] (version 0.1.4.0) Python package (https://github.com/py-why/causal-learn) to test the conditional independence between variables *X* and *Y*, given another variable *M* (denoted *X*  $\bot\!\!\!\!\bot $  *Y* | *M*) [20]. In the Fisher-z method, the correlation coefficient r between *X* and *Y* conditioned on *M* is converted to a z-score using this equation:


$$ w=\frac{1}{2}\ \ln \left(\frac{1+r}{1-r}\right) $$

$$ \sigma =\frac{1}{\sqrt{n-3}}\kern1.5em {z}_{score}=\frac{w-\mu }{\sigma } $$

The null hypothesis is *X* and *Y* are independent, conditioning on *M*. In the equation, *w* represents the Fisher-transformed correlation coefficient. $\mu$ is the mean of the Fisher-z transformed distribution under the null hypothesis, and n is the sample size. Then, the *P*-value is calculated by comparing the *z_score_* to the standard normal distribution.


$$ p=2\times \left(1-\Phi \left(\left|{z}_{score}\right|\right)\right) $$

$\varPhi \left(\left|{z}_{score}\right|\right)$ is the cumulative distribution function of the standard normal distribution. Since our objective is to identify independence (conditional on *M*), we retained pairs where we failed to reject the null hypothesis (*P* > .05).

### Search for the LR pairs used for cross-cell type communication

To identify LR pairs mediating cross-cell type communication, we applied the Fisher-z conditional independence test described above. A comprehensive dataset of 12,021 LR pairs was curated from multiple databases, including KEGG [21], Ramilowski [22], PPI_prediction [23], PPI_prediction_GO [24], and Guide2Pharmacology [25] databases. For each candidate DEG pair, the Fisher-z test was applied by conditioning on individual LR pairs from the curated database. Specifically, pseudo-bulk expression levels of T cell DEGs were defined as the causal variable (*X*), pseudo-bulk expression levels of non-T cell DEGs as the effect variable (*Y*), and the product of ligand and receptor pseudo-bulk expression levels as the conditioning variable (*M*), serving as a surrogate for LR interaction intensity. To ensure biological plausibility, additional co-expression constraints were imposed: ligands were required to be co-expressed with *X* in sender cells, and receptors were required to be co-expressed with *Y* in receiver cells, with a minimum Pearson correlation coefficient of 0.3 at the single-cell level.

### Group the results into GEMs to build the CCC networks

DEG pairs (DEG *X*, DEG *Y*) identified through the above analyses were grouped based on shared LR mediators to construct CCC networks. To ensure that the observed associations reflect LR-mediated communication rather than direct co-expression, results were filtered by applying a Pearson correlation threshold of <0.3 for the following comparisons: DEG *X* and DEG *Y*, DEG *X* and the ligand, DEG *X* and the LR complex, the receptor and DEG *Y*, and the LR complex and DEG *Y*. DEGs satisfying these criteria were grouped into GEMs, with co-regulated DEGs from sender cells forming GEM *X* and those from receiver cells forming GEM *Y*. Identified communication networks were validated through comprehensive literature review to assess concordance with established biological findings. Network visualizations were generated using BioRender (https://app.biorender.com/).

### Overview of contextual gene set analysis workflow

Contextual gene set analysis (cGSA) integrates experimental context into pathway analysis of DEGs using large language models (LLMs) [26]. By incorporating user-provided background information, cGSA enhances pathway relevance and assigns a contextual relevance score to each pathway. The pipeline consists of four steps: [1] detecting gene clusters using the EdMot [27] algorithm on DEG networks from STRING [2, 28] performing enrichment analysis via GSEA [3, 29] screening pathways with LLMs to refine relevance based on experimental context, and [4] summarizing pathways with LLMs to highlight key findings.

We applied cGSA to DEGs from the cell types in 31 breast cancer patients undergoing anti-PD-1 therapy. Cell-type-specific contexts were provided, incorporating treatment modality, cancer type, and treatment response status as contextual parameters. Pathway enrichment was performed with KEGG [21], WikiPathways [30], Reactome [31], MSigDB [32], and Panther [33], with human as the reference species.

## Results

### Profile of cell landscape under pre- and on-treatment conditions

**Figure 3:**
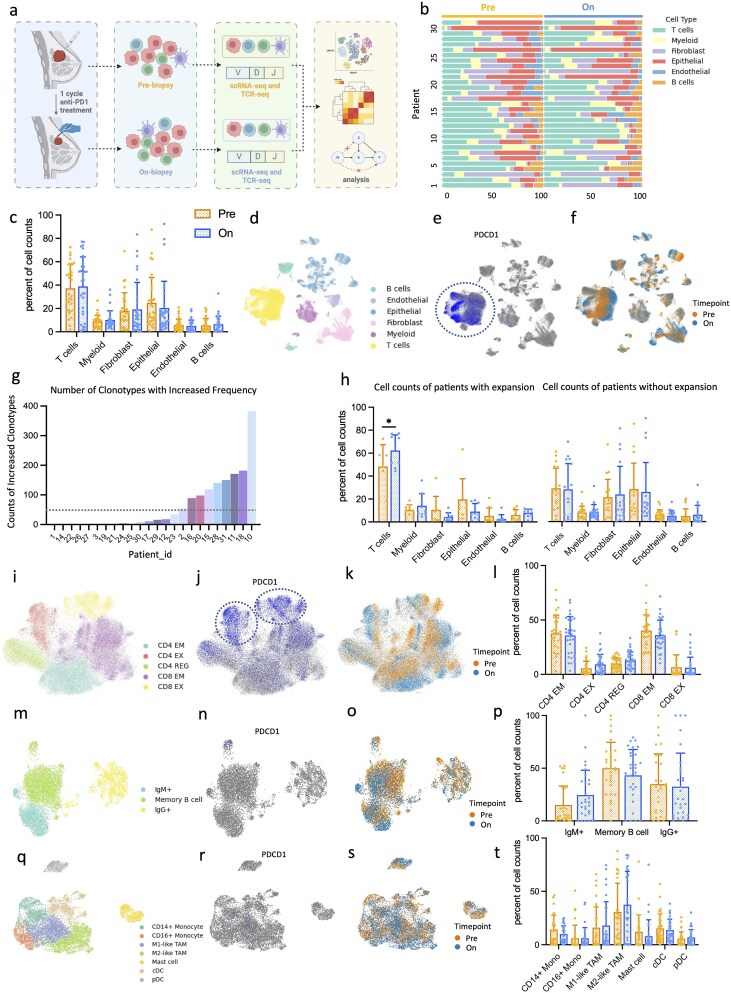
**Overview of experiment process and profile of pre- and on-treatment landscape. a**, Overview of the data collection and experiment design. Breast cancer biopsies are collected before and after one cycle of anti-PD-1 treatment (pembrolizumab) from 31 patients. Single-cell RNA sequencing data was collected from 168,970 cells. Then, a series of data analyses were performed to build a causal CCC network. **b**, Proportions of each cell type in each sample. **c**, Percentage of cell counts for each cell type in the samples before or after treatment. **d**, UMAP of 168,970 cells colored by cell types. **e**, UMAP colored by the expression level of *PDCD1*. **f**, UMAP colored by the timepoint of treatment. **g**, Number of clonotypes with increased frequency given TCR-seq data. **h**, Percentage of cell counts for each cell type in the samples with or without clonal expansion. **i-k**, UMAP of T cells colored by cell subtypes, expression level of *PDCD1, and* time point. **l**, Percentage of cell counts for T cell subtypes in the samples before or after treatment. **m-o**, UMAP maps of B cells colored by cell subtypes, timepoint, and expression level of PD-1. **p**, Percentage of cell counts for B cell subtypes in the samples before or after treatment. **q-s**, UMAP of myeloid cells colored by cell subtypes, timepoint, and expression level of PD-1. **t**, Percentage of cell counts for myeloid cell subtypes in the samples before or after treatment.

To evaluate the impact of anti-PD-1 therapy (pembrolizumab), we analyzed single-cell RNA-seq and TCR-seq data from a window-of-opportunity study reported by Bassez, *et al* [4]. This dataset comprised 168 970 cells from 31 patients, with samples collected under pre- and on-treatment conditions. Following quality control and dimensionality reduction (see Methods), we identified DEGs across cell types and employed causal inference methods to construct CCC networks (Fig. 3a).

We inspected the landscape of cells before and after treatment. The cellular compositions were highly heterogeneous across TMEs from 31 patients, potentially due to inter-tumor heterogeneity in TMEs or sampling different areas of tumors in different patients (Fig. 3b). However, the overall cell counts across various cell types remained largely unchanged throughout the treatment course (Fig. 3c). Cells grouped into distinct clusters corresponding to major lineages, including B, endothelial, epithelial, fibroblasts, myeloid, and T cells (Fig. 3d). For each cell type, a notable shift was observed on the UMAP map after treatment (Fig. 3f). Notably, T cells exhibited a more pronounced UMAP shift following treatment (Fig. 3f), whereas B cells and myeloid cells showed comparatively minor changes (Fig. 3o, s). This cell-type-specific pattern suggests that the observed T cell shift reflects treatment-induced transcriptomic changes rather than batch effects from data integration. Theoretically, the anti-PD-1 treatment only targets the cells expressing PD-1 [34]. *PDCD1* (encoding PD-1) expression was predominantly observed in T cells, with negligible expression in other cell types (Fig. 3e). These observations led us to hypothesize that treatment-induced transcriptomic changes in non-target cells arise through CCC networks initiated by *PDCD1*+ T cells.

Beyond examining overall cellular composition, we investigated T-cell clonal dynamics using TCR-seq data. Based on TCR-seq data, T-cell clonal expansion, defined as more than 50 TCR clones shared by more than 2 T cells (Fig. 3g), was observed in nine patients. Twenty patients showed no T-cell expansion. Two patients lacked TCR-seq data, leaving their clonal status undetermined (Fig. 3g). In patients with expansions (E), the composition of cell types changed more markedly after treatment compared to the patients with no expansions (NE), and T cells significantly increased after treatment (paired t-test, *P* < .05) (Fig. 3h).

T cells were clustered into five major subtypes, including CD4+ effector/memory cells (CD4 EM), CD4+ exhausted cells (CD4 EX), CD4+ regulatory cells (CD4 REG), CD8+ effector/memory cells (CD8 EM), and CD8+ exhausted cells (CD8 EX) (Fig. 3i). Although the T cell UMAP projection exhibited a distinct shift after treatment, the counts of T cell subtypes remained stable (Fig. 3l). *PDCD1* expression was broadly distributed among T cells, with a high concentration in CD4 EX and CD8 EX (Fig. 3j). B cells were divided into IgM+, Memory, and IgG+ cells (Fig. 3m), whereas myeloid cells are classified into CD14+ monocytes, CD16+ monocytes, M1-like tumor-associated macrophages (TAMs), M2-like TAMs, mast cells, plasmacytoid dendritic cells (pDC) and conventional dendritic cells (cDC) (Fig. 3q). Both B and myeloid cells have almost no *PDCD1* expression, and the subtype composition remained consistent after anti-PD-1 treatment. (Fig. 3n, p, r, t).

### Anti-PD-1 treatment induced broad gene expression changes in diverse cell populations

**Figure 4:**
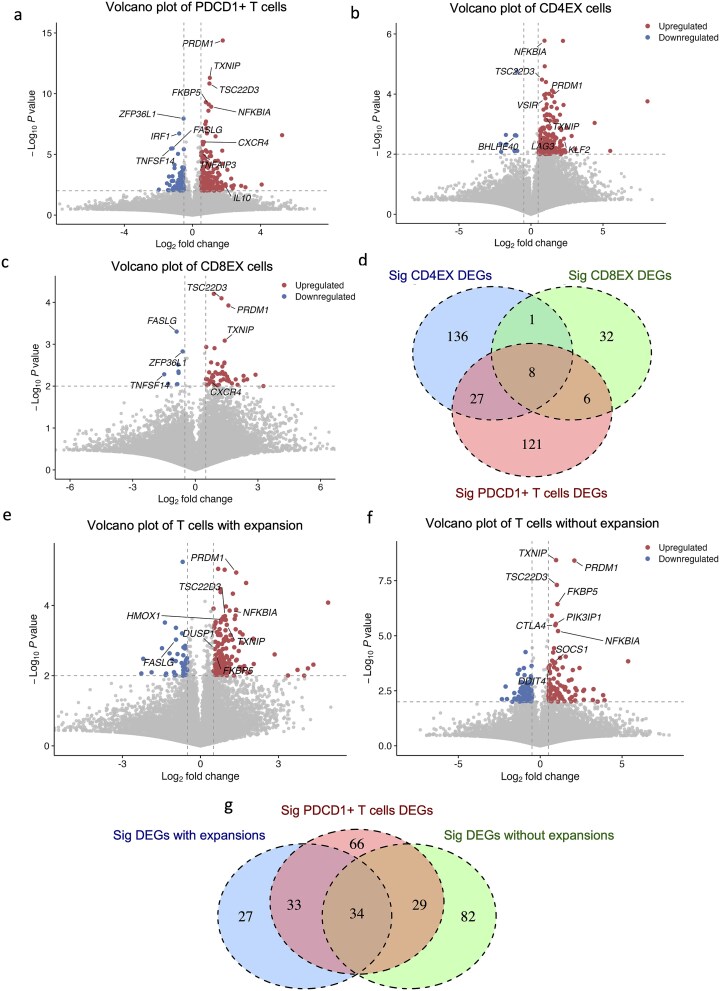
**Significant DEGs after the anti-PD-1 treatment in T cell subclusters. a**, Volcano plot showing DEGs in T cells expressing *PDCD1*, comparing before treatment and after treatment. Dots on the volcano plot: Gray, *P*-value is not significant; Red, *P* < .01 and log2 fold change is more than 0.5; Blue, *P* < .01 and log2 fold change is less than −0.5. *P*-values were obtained by the paired t-test. **b**, Volcano plot showing DEGs in CD4+ exhausted T cells comparing before treatment and after treatment. **c**, Volcano plot showing DEGs in CD8+ exhausted T cells comparing before treatment and after treatment. **d**, Venn diagram comparing significant DEGs across CD4+ T cells, CD8+ T cells, and all *PDCD1*+ T cells. Overlapping regions indicate genes shared between these groups, while non-overlapping regions represent group-specific DEGs. **e,** Volcano plot showing DEGs in T cells from E tumors comparing before treatment and after treatment. **f,** Volcano plot showing DEGs in T cells from NE tumors, comparing before treatment and after treatment. **g**, Venn diagram comparing significant DEGs of T cells in E tumors, NE tumors, and all T cells.

We analyzed DEGs in *PDCD1*-expressing T cells (Fig. 4a). The top five upregulated DEGs ranked by *P*-value were *PRDM1*, *TXNIP*, *TSC22D3*, *FKBP5*, and *NFKBIA*, while the top five downregulated DEGs were *ZFP36L1*, *IRF1*, *FASLG*, *TNFSF14,* and *IER2*. Notably, *PRDM1*, a key transcription factor in T and B cell differentiation, showed treatment-induced upregulation alongside altered expression of its known downstream targets, including *IL10*, *IL2*, and *BATF* [35]. Furthermore, multiple NF-κB-regulated genes (*NFKBIA*, *CXCR4*, *FASLG*, and *TNFAIP3*) [36] were differentially expressed following anti-PD-1 treatment, indicating modulation of this pathway.

To investigate subpopulation-specific responses, we compared DEGs between CD4 EX and CD8 EX cells, both of which express high levels of *PDCD1* relative to other T cell subtypes (Fig. 4b and c). These two subpopulations exhibited partially overlapping but distinct DEG profiles (Fig. 4d). Shared DEGs included *PRDM1*, *TXNIP*, and *TSC22D3*, while subtype-specific DEGs were also identified: *VSIR*, *KLF2*, and *BHLHE40* in CD4 EX T cells, and *FASLG*, *CXCR4*, and *TNFSF14* in CD8 EX T cells.

T cells from E and NE tumors exhibited differential responses to anti-PD-1 treatment. Although both tumor subgroups shared a subset of DEGs, they also displayed distinct transcriptional signatures, indicating divergent responses to immunotherapy depending on TME context (Fig. 4e–g). In E tumors, several upregulated DEGs were associated with anti-tumor immune responses, including *DUSP1* [37] and *HMOX1* [38].

### Discovering potential cell–cell communication between *PDCD1*+ T cells and non-T cells in TME through causal analysis

**Figure 5:**
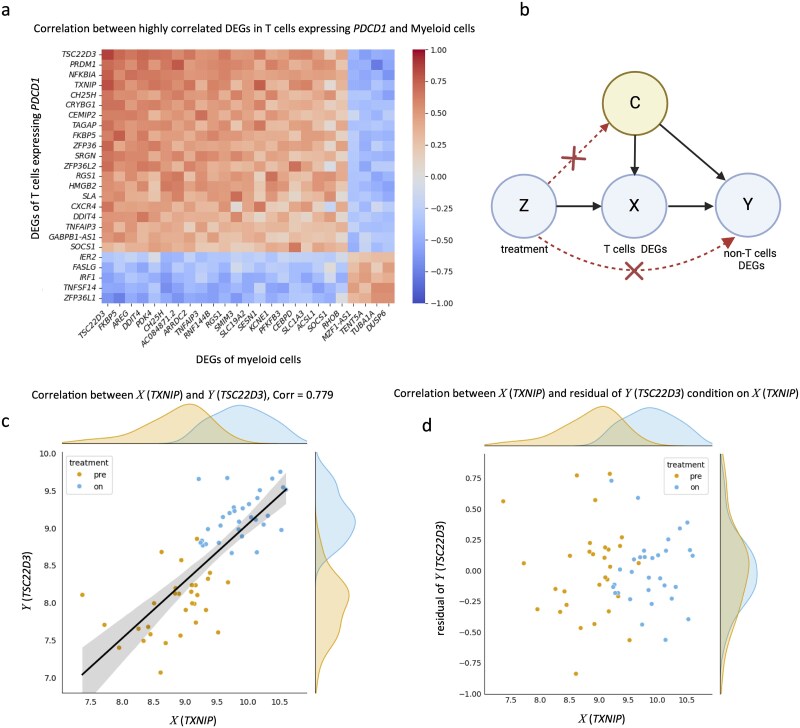
**Significant DEG correlation between specific T cell clusters and other cell type clusters. a**, Heatmap of highly correlated DEGs of T cells expressing *PDCD1* and myeloid cells. **b**, Causal graph of application of the instrumental variable. **c**, The scatter plot showing the correlation between *X* (*TXNIP*) and *Y* (*TSC22D3*). Pearson correlation = 0.779. **d**, the scatter plot showing the correlation between *X* (*TXNIP*) and the residual of *Y* (*TSC22D3*) conditioned on *X* (*TXNIP*).

Our analyses revealed substantial DEGs among non-T cells in response to anti-PD-1 treatment. Many of these DEGs exhibited strong correlations with T-cell DEGs, suggesting potential CCC (Fig. 5a). Notably, certain DEG pairs between *PDCD1*+ T cells and myeloid cells showed high correlations (|r| > 0.7), indicating that anti-PD-1 therapy induced coordinated transcriptional changes across multiple cell types in the TME.

Since myeloid cells do not express *PDCD1*, anti-PD-1 treatment is unlikely to affect them directly. Therefore, the strong correlation between T cell and myeloid DEGs likely reflects CCC, whereby treatment-induced changes in T cells causally influence myeloid cell states. To test this hypothesis while accounting for potential confounders, we applied IV analysis [39].

As an illustrative example, *TXNIP* (*PDCD1*+ T cell DEG) and *TSC22D3* (myeloid DEG) exhibited a strong correlation (r = 0.779), with both showing bimodal distributions associated with treatment condition (Fig. 5c). After conditioning on *TXNIP* expression, *TSC22D3* expression became independent of treatment status (Fig. 5d), supporting the hypothesis that anti-PD-1 treatment influences myeloid gene expression through CCC with *PDCD1*+ T cells. The molecular mechanisms underlying this CCC channel are discussed in a later section.

Systematic IV analysis across all cell type pairs (Supplementary Table S1) revealed that *PDCD1*+ T cell DEGs exhibited stronger causal relationships with myeloid, endothelial, and fibroblast DEGs compared to B and epithelial cell DEGs (Supplemental Fig. 2a and b and Supplemental Fig. 3a and b). Similar patterns were observed at the subtype level, including between CD4 EX and monocytes (Supplemental Fig. 4a and b).

### Building CCC networks by searching for potential ligand-receptor interactions

**Figure 6:**
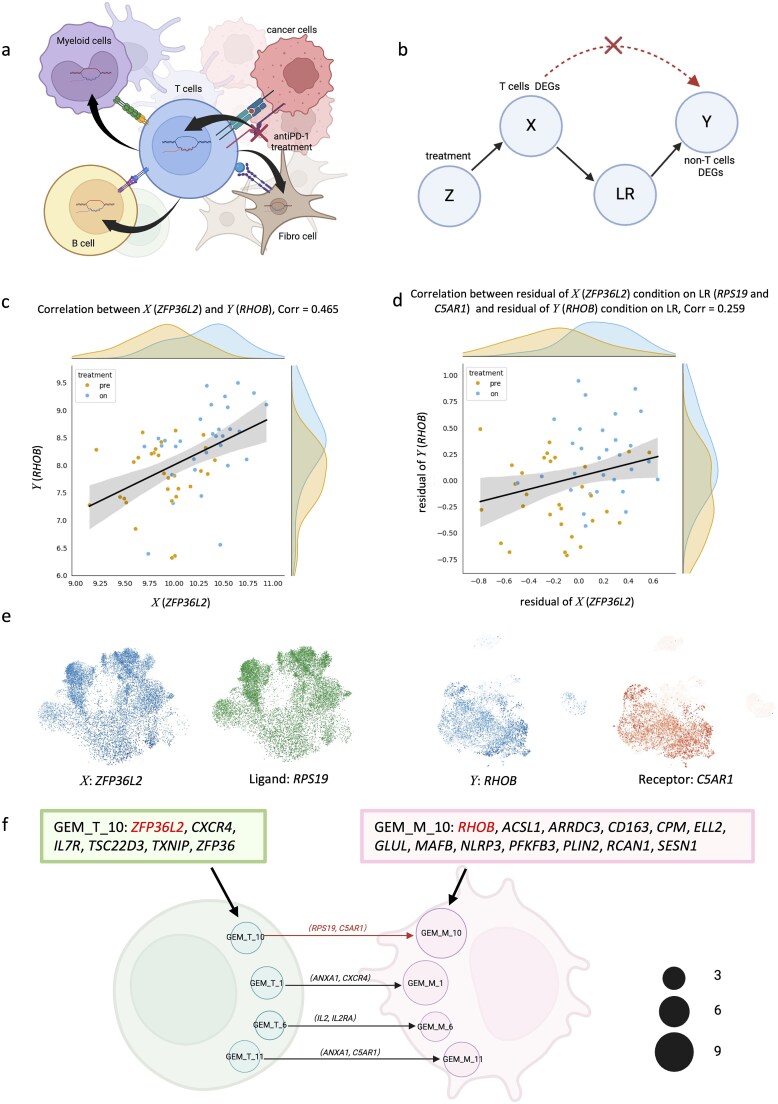
**Validation of the significant DEG correlations between T and non-T cells using causal inference methods. a**, A simplified illustration of the CCC network after anti-PD-1 treatment. **b**, The causal graph of application of the conditional independence test. **c**, The scatter plot depicting the correlation between *X* (*ZFP36L2*) and *Y* (*RHOB*). Pearson correlation = 0.465. **d**, The scatter plot depicting the correlation between residual of *X* (*ZFP36L2*) conditioned on *Y* (*RHOB*) and residual of *Y* (*RHOB*) conditioned on the ligand (*RPS19*) and receptor (*C5AR1*). Pearson correlation = 0.259. **e**, UMAP plots of *X* (*ZFP36L2*) and ligand (*RPS19*) in T cells, and UMAP plots of *Y* (*RHOB*) and receptor (*C5AR1*) in myeloid cells. **f**, An illustration of the CCC network between T cells and myeloid cells, represented using GEMs and ligand-receptor pairs. The size of each circle reflects the number of DEGs within each GEM. The composition of DEGs in GEM_T_10 and GEM_M_10 is highlighted in this figure.

Having established potential causal relationships between the DEGs from *PDCD1*+ T cells and those from non-T cells, we next sought to identify the ligand-receptor (LR) pairs mediating these interactions (Fig. 6a). Using conditional independence tests, we searched for LR pairs that, when conditioned upon, rendered the correlated DEG pairs independent, indicating potential signal transduction (Fig. 6b). As an example, *ZFP36L2* and *RHOB* were significantly correlated (r = 0.465). Notably, *RPS19* (ligand) and *ZFP36L2* are co-expressed in *PDCD1*+ T cells, while *C5AR1* (receptor) and *RHOB* are co-expressed in macrophages (Fig. 6e). After conditioning on RPS19-C5AR1 interaction, the correlation between *ZFP36L2* and *RHOB* decreased substantially (r < 0.3) (Fig. 6d), suggesting that RPS19-C5AR1 mediates signal transduction between *PDCD1*+ T cells and myeloid cells.

As illustrated in Fig. 1, treatment-induced pathway activation in T cells leads to coordinated expression changes across multiple DEGs (GEM *X*), which subsequently influence non-T cells through LR-mediated communication, resulting in correlated DEG modules (GEM *Y*). Therefore, an LR pair may be detected to be conditionally independent between multiple members of GEM *X* and GEM *Y*. In other words, identifying an LR pair that likely mediates signaling between multiple pairs of DEGs across cell types serves as a means to search GEMs. We identified such GEMs in *PDCD1*+ T cells and corresponding non-T cells that share common LR mediators, defining these LR pairs as CCC channels.

Systematic screening identified multiple CCC channels (Supplementary Fig. 5a). For example, the *RPS19-C5AR1* LR pair potentially mediates correlated expression between GEM_T_10 (T cell module) and GEM_M_10 (myeloid module) (Fig. 6f). These modules showed co-expression with their respective ligand and receptor on UMAP plots (Supplemental Fig. 5b). The correlation between GEM_T_10 and GEM_M_10 decreased from r = 0.464 (*P* < .001) to r = 0.238 after conditioning on RPS19-C5AR1 (Supplemental Fig. 5c). To characterize the biological functions of these CCC channels, we also performed pathway analysis using cGSA [26], which revealed distinct communication patterns between E and NE tumors (Supplemental Fig. 6a, b). Notably, in NE tumors, we identified multiple CCC channels connecting *PDCD1*+ T cell DEGs to myeloid DEGs involved in fatty acid metabolism. Given that fatty acid metabolism in myeloid cells promotes tumorigenesis and treatment resistance [40, 41], this CCC pattern may contribute to anti-PD-1 resistance mechanisms in NE tumors.

### Exhausted T cell subsets engage distinct CCC networks with TAMs associated with treatment response

**Figure 7:**
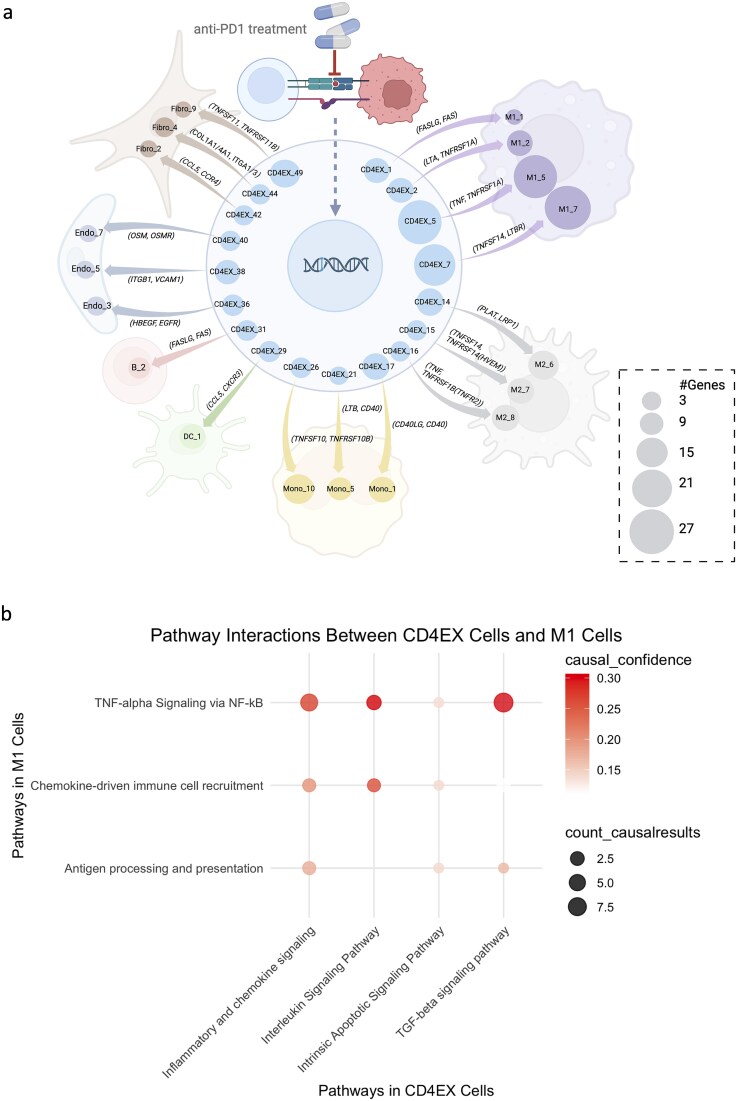
**Summary of CCC network of CD4+ exhausted cells with non-T cells in TME. a**, The CCC network of CD4+ exhausted cells with non-T cells, including monocytes, B cells, dendritic cells, M1-like TAMs, M2-like TAMs, fibroblasts, and endothelial cells. The size of each circle reflects the number of DEGs within each GEM. **b**, A dot plot of pathway interactions between CD4+ exhausted T cells and M1-like TAMs. The pathways are enriched using the cGSA method. The size of the dots indicates the count of causally related DEG pairs *X*-*Y* (*X* from T cells and *Y* from myeloid cells). The color intensity of the dots indicates the significance of the causal relationship, which is the *P*-value of the Fisher-z CIT test.

**Figure 8:**
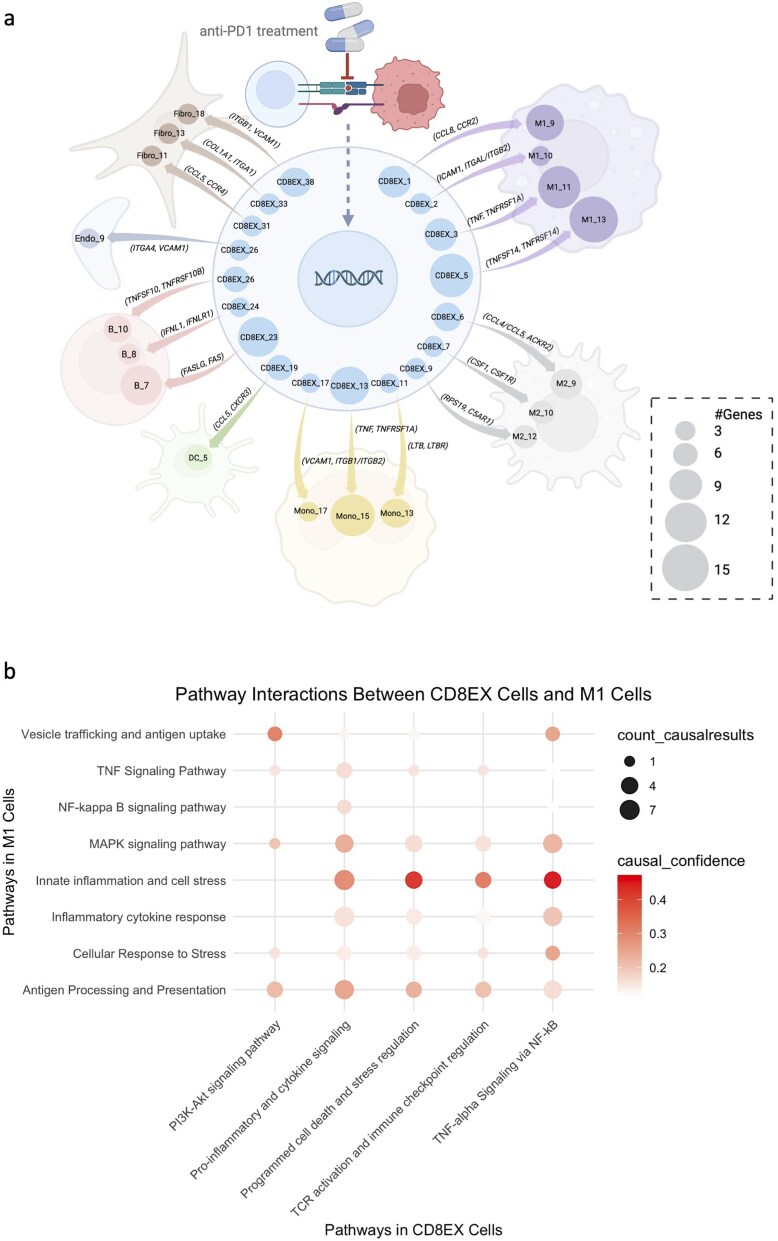
**Summary of CCC network of CD8+ exhausted cells with non-T cells in TME. a**, The CCC network of CD8+ exhausted cells with non-T cells. **b**, A dot plot of pathway interactions between CD8+ exhausted T cells and M1-like TAMs.

CD4 EX and CD8 EX utilized distinct ligand-receptor interactions to communicate with other cell types (Supplementary Table S2 and S3). Tumor-associated macrophages (TAMs), comprising anti-tumor M1-like and pro-tumor M2-like subsets, are key regulators of the TME and significantly influence anti-PD-1 therapy efficacy [42]. Our findings indicate that CD4 EX communicated with M1-like TAMs to enhance their anti-tumor function through ligand-receptor interactions, including TNF-TNFRSF14 [43] and TNFSF14-LTBR [44] (Fig. 7a). Integrating the result of cGSA pathway enrichment analysis and causal inference analysis, we observed that the activation of signaling pathways in CD4 EX T cells is causally linked to the activation of TNF-alpha signaling via NF-kB pathway in M1 TAMs through ligand-receptors (Fig. 7b). Similarly, CD8 EX engaged in crosstalk with M1-like TAMs through TNF-TNFRSF1A [45], ICAM1-ITGAL/ITGB2 [46, 47], and CCL8-CCR2 [48] to create an anti-tumor TME (Fig. 8a and b).

Notably, 89% of M1-like TAMs originated from E tumors, whereas 90% of M2-like TAMs derived from NE tumors. Correspondingly, CCC between CD4 EX/CD8 EX and M1-like TAMs was predominantly observed in E tumors, while CCC with M2-like TAMs was enriched in NE tumors. Given that TCR clonotype expansion serves as a biomarker of anti-PD-1 responsiveness [4], our findings suggest that the CCC patterns from CD4/CD8 EX T cells to M1 TAMs are highly enriched in E tumors, providing mechanistic insights into anti-PD-1 efficacy. Conversely, CCC between CD4/CD8 EX T cells and M2-like TAMs may elucidate how pro-tumor pathways are activated in NE tumors, contributing to treatment resistance. These CCC interactions represent potential therapeutic targets for enhancing responses to immune checkpoint blockade [49].

In NE tumors, CD4 EX/CD8 EX interactions with M2-like TAMs involved several receptors currently under clinical investigation, including TNFRSF1B (TNFR2) [50], CSF1R [51, 52], and C5AR1 [53, 54]. The identification of these established therapeutic targets validates our framework’s ability to discover clinically relevant CCC channels. Beyond known targets, our analysis identified additional LR interactions with therapeutic potential. TNFRSF14 (HVEM) [55], CSF2RB [56], and LRP1 [57] have been implicated in the regulation of M2-like TAMs and immune responses following anti-PD-1 therapy. From the causal inference perspective, our CCC result shows that these receptors can be the next promising candidates for combination immunotherapy strategies to improve treatment efficacy.

## Discussion

While numerous studies have demonstrated that TME cellular components communicate and influence each other [58–60], few studies have examined CCC through a causal lens [61]. In this study, we developed scIVCCC, a causal inference framework that leverages anti-PD-1 treatment as an instrumental variable to distinguish genuine CCC from confounded associations. Applying this framework to single-cell RNA-seq data from breast cancer patients, we identified treatment-responsive CCC networks linking *PDCD1*+ T cells to non-T cell populations, with particular emphasis on exhausted T cell interactions with TAM subsets that distinguish treatment-responsive from non-responsive tumors.

Our causal framework offers distinct advantages over existing CCC inference methods. Current state-of-the-art tools such as CellChat [62], CellPhoneDB [12], and NicheNet [11] excel at cataloging potential LR interactions without requiring perturbation conditions, enabling rapid exploration of CCC landscapes. However, these association-based approaches cannot distinguish whether detected correlations reflect true causal communication or arise from confounding factors such as shared microenvironmental signals or patient-specific effects. In a translational context, relying on simple correlations poses a risk of targeting downstream effects rather than upstream drivers. Unlike association-based methods, our IV-based approach explicitly accounts for unobserved confounders, such as shared microenvironmental signals, patient-specific immune states, and batch effects, ensuring that identified CCC patterns represent genuine intercellular signaling induced by the therapy itself, rather than spurious correlations arising from common upstream factors.

Beyond filtering confounders, our IV-based approach establishes causal directionality and, through identification of gene expression modules co-regulated with LR pairs, provides mechanistic insights into the signaling pathways mediating CCC. However, our framework has inherent limitations. First, it requires a perturbation condition that satisfies instrumental variable assumptions, limiting applicability to observational datasets without such interventions. Second, the approach focuses on treatment-responsive CCC channels and may not capture constitutive communication patterns present in both conditions. Third, although we did not incorporate spatial transcriptomics data in the current study due to the limited availability of breast cancer spatial transcriptomic datasets with paired samples before and after anti-PD-1 treatment, spatial information would enhance CCC analysis by confirming cellular proximity. As such datasets become available, integrating spatial context with causal inference will be an important future direction. In summary, causal inference provides a principled framework for distinguishing genuine communication from spurious associations, yielding more actionable hypotheses for experimental validation.

The identification of CCC channels between exhausted T cells and TAM subsets has direct translational implications. Our framework successfully nominated receptors already under clinical investigation, validating its ability to identify therapeutically relevant targets. Importantly, the distinct CCC patterns observed between E and NE tumors suggest that these networks may serve as biomarkers for predicting treatment response and as a basis for designing rational combination therapies. Furthermore, causal inference methods can simulate the effects of perturbing specific CCC channels, positioning this framework as a tool for computational drug discovery.

Notably, several identified receptors already have agents in clinical development (e.g. C5AR1 antagonists [54], CSF1R inhibitors [52]), providing opportunities for combination trials with anti-PD-1 therapy. Such validation is essential before these causal biomarkers can be utilized for patient stratification or therapeutic targeting.

In summary, we demonstrated the utility of causal inference methods to investigate CCC in breast cancer patients receiving anti-PD-1 therapy. This framework can be generalized to other single-cell RNA-seq datasets across various cancer types and treatment modalities. Future integration of causal inference with spatial transcriptomics would substantially enhance precision by incorporating cellular proximity information, enabling refined network reconstruction and potentially revealing spatially constrained therapeutic targets. Ultimately, this causal approach offers a robust strategy for identifying actionable therapeutic targets to improve clinical outcomes in cancer immunotherapy.

**Key Points**

- Anti-PD-1 therapy induces widespread transcriptional reprogramming across diverse cell populations in the breast cancer tumor microenvironment, extending beyond *PDCD1*+ T cells.
- The scIVCCC framework distinguishes causal relationships from statistical correlations, revealing distinct, treatment-driven communication networks linking exhausted T cells to M1-like and M2-like tumor-associated macrophages.
- We identify key signaling axes, including TNF–TNFRSF1A and ICAM1–ITGAL/ITGB2, as potential drivers of anti-tumor M1-like tumor-associated macrophage activation.
- Immunosuppressive signals, including CSF1–CSF1R, RPS19–C5AR1, and TNFSF14–HVEM, potentially drive M2-like tumor-associated macrophage polarization and resistance to anti-PD-1 treatment.
- Targeting key receptors involved in cell–cell communication, such as C5AR1, HVEM, and TNFR2, may enhance anti-PD-1 treatment efficacy.

## Supplementary Material

Supplementary_material_bbag139

## Data Availability

The single-cell RNA-seq data from pre- and on-treatment breast cancer samples are available through the European Genome-phenome Archive (EGA) under accession number EGAS00001004809.

## Code availability

The source code for scIVCCC is available at https://github.com/pikapika-qiu/scIVCCC.git.
