# Supplementary material for: Causal modeling reveals cell–cell communication dynamics in the tumor microenvironment during anti-PD-1 therapy in breast cancer patients
Source: Brief Bioinform. 2026 Apr 19;27(2):bbag139. doi: 10.1093/bib/bbag139 (PMC13092271; doi:10.1093/bib/bbag139)
Supplement: Supplementary_material_bbag139 [file supplementary_material_bbag139.zip › Supplement_revised_complete.docx]

**Supplemental Figure 1.** **Annotation of cell types.**

**a**, A dot plot of the annotation of major cell types. **b**, A dot plot of the annotation of T cell subtypes. **c**, A dot plot of the annotation of myeloid cell subtypes.

**Supplemental Figure 2.** **Correlation between the DEGs of *PDCD1*⁺ T cells and the DEGs of endothelial and B cells.**

**a,** Heatmap displaying the correlation coefficients between DEGs in *PDCD1*+ T cells (y-axis) and DEGs in endothelial cells (x-axis). **b,** Heatmap displaying the correlation coefficients between DEGs in *PDCD1*+ T cells (y-axis) and DEGs in B cells (x-axis). Values within tiles in the heatmap represent correlation coefficients (top) and significance statistics defined as -log_10_(p-value) (bottom). The color scale indicates the strength of correlation (red: positive; blue: negative).

**Supplemental Figure 3.** **Correlation between the DEGs of *PDCD1*+ T cells and the DEGs of fibroblast and epithelial cells.**

**a**, Heatmap displaying the correlation between DEGs identified in *PDCD1*+ T cells and DEGs identified in fibroblast cells. **b,** Heatmap displaying the correlation between DEGs identified in *PDCD1*+ T cells and DEGs identified in epithelial cells. Values in heatmap tiles represent the Pearson correlation coefficient (top) and the statistical significance defined as -log_10_(p-value) (bottom). The color scale indicates the strength of correlation (red: positive; blue: negative).


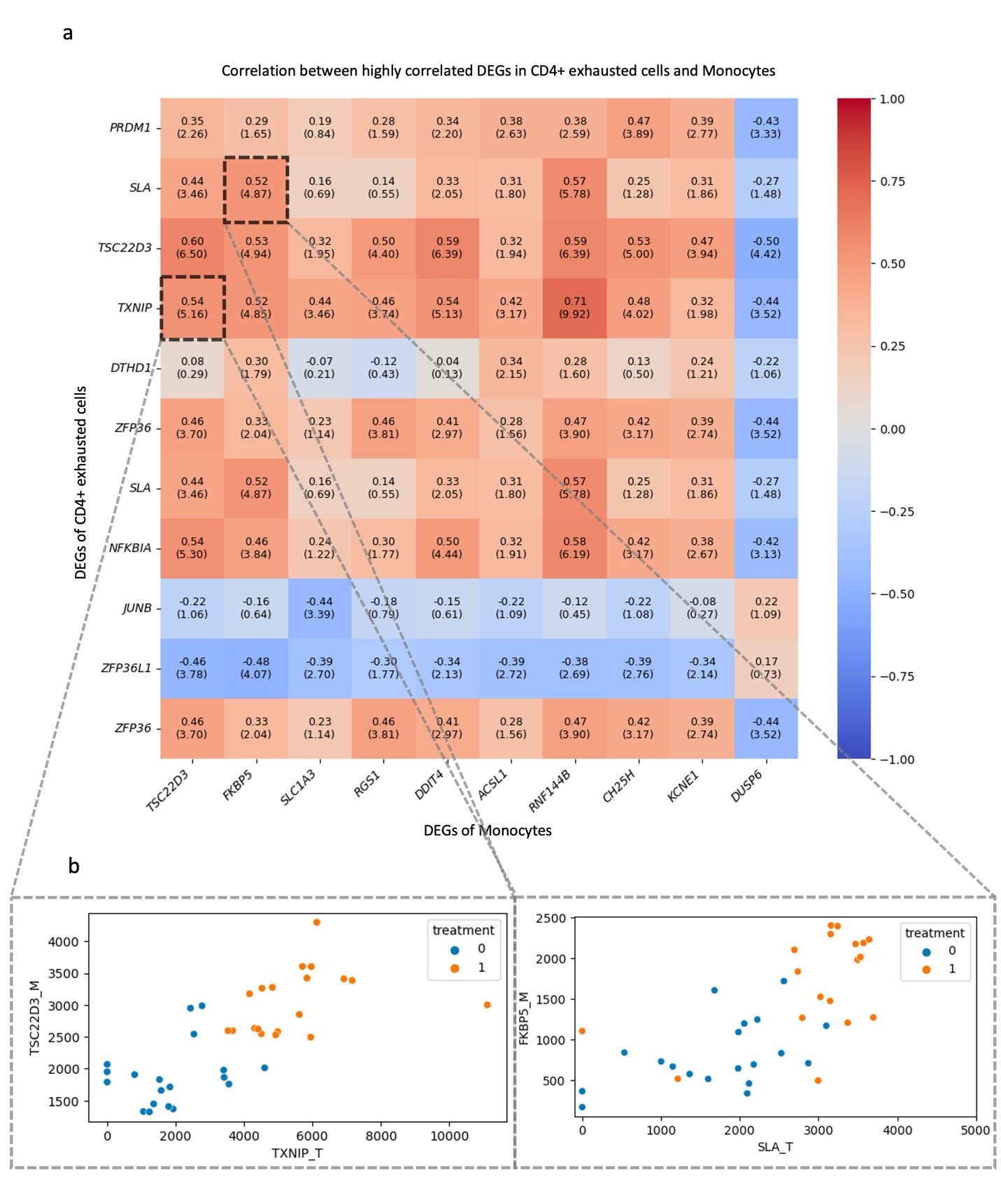


**Supplemental Figure 4.** **Significant DEG correlation between CD4+ exhausted T cells and Monocytes.**

**a,** Heatmap displaying the correlation between DEGs identified in CD4+ exhausted T cells and DEGs identified in monocytes. **b,** Scatter plots illustrating the correlation between specific gene pairs: *TXNIP* (T cells) vs. *TSC22D3* (Monocytes) (left) and *SLA* (T cells) vs. *FKBP5* (Monocytes) (right). Values in tiles in the heatmap represent the Pearson correlation coefficient (top) and the statistical significance defined as -log_10_(p-value) (bottom). The color scale indicates the strength of correlation (red: positive; blue: negative).

**Supplemental Figure 5.** **Summary of significant communication between *PDCD1*+ T cells and other cells in TME.**

**a**, The CCC network of *PDCD1*+ T cells with non-T cells, including Myeloid cells (M), B cells (B), Fibroblasts (Fibro), and endothelial cells (Endo). The size of each circle reflects the number of DEGs within each GEM. **b**, UMAP plots of GEM_T_10 and ligand (*RPS19*) in *PDCD1*+ T cells, and UMAP plots of GEM_M_10 and receptor (*C5AR1*) in myeloid cells. **c**, Scatter plots showing the correlation between GEM_T_10 and GEM_M_10 before (left, r = 0.464) and after (right, r = 0.238) conditioning on the RPS19–C5AR1 ligand-receptor interaction. The reduction in correlation after conditioning supports that RPS19–C5AR1 mediates the communication between these gene expression modules.


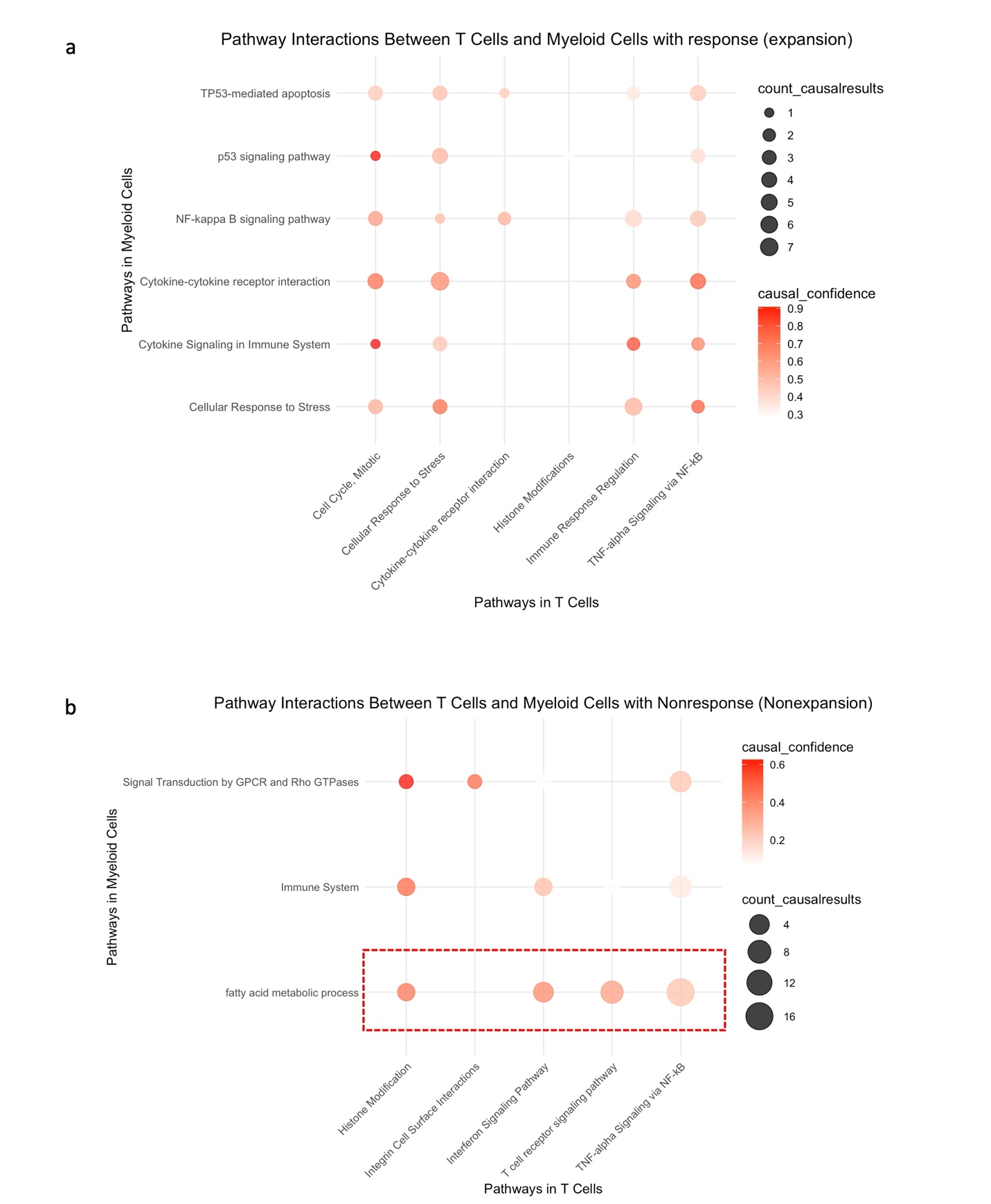


**Supplemental Figure 6.** **Communication between *PDCD1*+ T cells and myeloid cells in patients with expansion and without expansion.**

**a**, A dot plot of pathway interactions between *PDCD1*+ T cells (x-axis) and Myeloid cells (y-axis) in the patients with TCR clonotype expansion. The size of the dots indicates the count of causally related DEG pairs *X-Y* (*X* from T cells and *Y* from Myeloid cells). The color of the dots indicates the significance of the causal relationship, which is the p-value of the CIT test. **b**, A dot plot of pathway interactions between *PDCD1*+ T cells (x-axis) and Myeloid cells (y-axis) in the patients without TCR clonotype expansion.
